# Supplementary material for: Sparse haplotype-based fine-scale local ancestry inference at scale reveals recent selection on immune responses
Source: Nat Commun. 2025 Mar 20;16:2742. doi: 10.1038/s41467-025-57601-3 (PMC11926123; doi:10.1038/s41467-025-57601-3)
Supplement: Supplementary file 2 — Description of Additional Supplementary Files [file 41467_2025_57601_MOESM2_ESM.pdf]

# Description of Additional Supplementary Files

File Name: Supplementary Data 1
Description: Summary statistics for 10,000 UK Biobank individuals with self-reported British ethnic background at each SNP, including LDAS, AAS, and average probabilities of 26 1000GP populations. The -log10 scale of p-values for LDAS and AAS are computed from one-sided normality test and two-sided chi-squared test, respectively. The LDAS of SNPs which are removed in quality control are displayed as `NA'.

File Name: Supplementary Data 2
Description: Summary statistics for 12,713 UK Biobank individuals with self-reported Irish ethnic background at each SNP, including LDAS, AAS, and average probabilities of 26 1000GP populations. The -log10 scale of p-values for LDAS and AAS are computed from one-sided normality test and two-sided chi-squared test, respectively. The LDAS of SNPs which are removed in quality control are displayed as `NA'.

File Name: Supplementary Data 3
Description: Summary statistics for 3,203 UK Biobank individuals with self-reported African ethnic background at each SNP, including LDAS, AAS, and average probabilities of 26 1000GP populations. The -log10 scale of p-values for LDAS and AAS are computed from one-sided normality test and two-sided chi-squared test, respectively. The LDAS of SNPs which are removed in quality control are displayed as `NA'.

File Name: Supplementary Data 4
Description: Summary statistics for 4,279 UK Biobank individuals with self-reported Caribbean ethnic background at each SNP, including LDAS, AAS, and average probabilities of 26 1000GP populations. The -log10 scale of p-values for LDAS and AAS are computed from one-sided normality test and two-sided chi-squared test, respectively. The LDAS of SNPs which are removed in quality control are displayed as `NA'.

File Name: Supplementary Data 5
Description: Summary statistics for 5,660 UK Biobank individuals with self-reported Indian ethnic background at each SNP, including LDAS, AAS, and average probabilities of 26 1000GP populations. The -log10 scale of p-values for LDAS and AAS are computed from one-sided normality test and two-sided chi-squared test, respectively. The LDAS of SNPs which are removed in quality control are displayed as `NA'.

File Name: Supplementary Data 6
Description: Summary statistics for 1,747 UK Biobank individuals with self-reported Pakistani ethnic background at each SNP, including LDAS, AAS, and average probabilities of 26 1000GP populations. The -log10 scale of p-values for LDAS and AAS are computed from one-sided normality test and two-sided chi-squared test, respectively. The LDAS of SNPs which are removed in quality control are displayed as `NA'.

File Name: Supplementary Data 7

Description: Summary statistics for 1,503 UK Biobank individuals with self-reported Chinese ethnic background at each SNP, including LDAS, AAS, and average probabilities of 26 1000GP populations. The -log10 scale of p-values for LDAS and AAS are computed from one-sided normality test and two-sided chi-squared test, respectively. The LDAS of SNPs which are removed in quality control are displayed as `NA'.

File Name: Supplementary Data 8

Description: SNPs with shared LDAS or AAS signals. The structural variation information in 1000GP for those SNPs reported by Sudmant et al. (2015) is also listed.

Reference: Sudmant, P. H. et al. An integrated map of structural variation in 2,504 human genomes. Nature 526, 75–81 (2015).
