# Supplementary material for: Sparse haplotype-based fine-scale local ancestry inference at scale reveals recent selection on immune responses
Source: Nat Commun. 2025 Mar 20;16:2742. doi: 10.1038/s41467-025-57601-3 (PMC11926123; doi:10.1038/s41467-025-57601-3)
Supplement: Supplementary file 11 — Reporting Summary [file 41467_2025_57601_MOESM11_ESM.pdf]

nature portfolio

Corresponding author(s): Yaoling Yang
Daniel John Lawson

Last updated by author(s): 13/1/25

Reporting Summary

Nature Portfolio wishes to improve the reproducibility of the work that we publish. This form provides structure for consistency and transparency in reporting. For further information on Nature Portfolio policies, see our Editorial Policies and the Editorial Policy Checklist.

Statistics

For all statistical analyses, confirm that the following items are present in the figure legend, table legend, main text, or Methods section.

n/a	Confirmed	
☐	☒	The exact sample size (*n*) for each experimental group/condition, given as a discrete number and unit of measurement
☐	☒	A statement on whether measurements were taken from distinct samples or whether the same sample was measured repeatedly
☐	☒	The statistical test(s) used AND whether they are one- or two-sided<br>*Only common tests should be described solely by name; describe more complex techniques in the Methods section.*
☐	☒	A description of all covariates tested
☐	☒	A description of any assumptions or corrections, such as tests of normality and adjustment for multiple comparisons
☐	☒	A full description of the statistical parameters including central tendency (e.g. means) or other basic estimates (e.g. regression coefficient) AND variation (e.g. standard deviation) or associated estimates of uncertainty (e.g. confidence intervals)
☐	☒	For null hypothesis testing, the test statistic (e.g. *F*, *t*, *r*) with confidence intervals, effect sizes, degrees of freedom and *P* value noted<br>*Give P values as exact values whenever suitable.*
☒	☐	For Bayesian analysis, information on the choice of priors and Markov chain Monte Carlo settings
☒	☐	For hierarchical and complex designs, identification of the appropriate level for tests and full reporting of outcomes
☐	☒	Estimates of effect sizes (e.g. Cohen's *d*, Pearson's *r*), indicating how they were calculated

Our web collection on statistics for biologists contains articles on many of the points above.

Software and code

Policy information about availability of computer code

Data collection	No software was used
Data analysis	ChromoPainter<br>PBWT<br>FLARE<br>RFMix<br>MOSAIC<br>Neural-ADMIXTURE<br>gprofiler2<br>sparsesvd<br>nnls<br>bcftools<br>QCTools<br>The C++ code for SparsePainter is available on GitHub at https://github.com/YaolingYang/SparsePainter, and the website for SparsePainter is at https://sparsepainter.github.io/. PBWTpaint is available on GitHub at https://github.com/richarddurbin/pbwt.

For manuscripts utilizing custom algorithms or software that are central to the research but not yet described in published literature, software must be made available to editors and reviewers. We strongly encourage code deposition in a community repository (e.g. GitHub). See the Nature Portfolio guidelines for submitting code & software for further information.

Data

Policy information about availability of data

All manuscripts must include a data availability statement. This statement should provide the following information, where applicable:

- Accession codes, unique identifiers, or web links for publicly available datasets
- A description of any restrictions on data availability
- For clinical datasets or third party data, please ensure that the statement adheres to our policy

The phased 1000 Genomes Project data build GRCh37/hg19 are available at https://bochet.gcc.biostat.washington.edu/beagle/1000_Genomes_phase3_v5a/b37.vcf/. The genetic map build GRCh37/hg19 is available from https://bochet.gcc.biostat.washington.edu/beagle/genetic_maps. The UK Biobank data can be accessed by approved researchers through https://www.ukbiobank.ac.uk. We used the UK Biobank data under project 81499. The UK map data are available at https://gadm.org.

Research involving human participants, their data, or biological material

Policy information about studies with human participants or human data. See also policy information about sex, gender (identity/presentation), and sexual orientation and race, ethnicity and racism.

Reporting on sex and gender	Sex-based analyses were not performed.
Reporting on race, ethnicity, or other socially relevant groupings	Our study included 7 self-reported ethnic groups from the UK Biobank: British, Irish, Indian, Pakistani, African, Caribbean, Chinese. We investigated the genes with LDAS and AAS signals which are shared between all these 7 self-reported ethnic groups.
Population characteristics	N/A
Recruitment	N/A
Ethics oversight	We used the UK Biobank data under project 81499.

Note that full information on the approval of the study protocol must also be provided in the manuscript.

Field-specific reporting

Please select the one below that is the best fit for your research. If you are not sure, read the appropriate sections before making your selection.

- [ ] Life sciences
- [ ] Behavioural & social sciences
- [x] Ecological, evolutionary & environmental sciences

For a reference copy of the document with all sections, see nature.com/documents/nr-reporting-summary-flat.pdf

Ecological, evolutionary & environmental sciences study design

All studies must disclose on these points even when the disclosure is negative.

Study description	We present PBWTpaint and SparsePainter which are used for efficient local ancestry inference. We show haplotypes better represent ancestries than principal components, whilst linkage-disequilibrium of ancestry identifies signals of recent changes to population-specific selection for many genomic regions associated with immune responses.
Research sample	Our samples were taken from the UK Biobank and 1000 Genomes Project. All the samples are included in our study.
Sampling strategy	We randomly sampled 10000 individuals from self-reported British ethnic background in the UK Biobank, and used all the individuals from the other 6 ethnicities we investigate. The sampling strategy ensured the sample size in each ethnicities do not differ substantially.
Data collection	Data were collected by the UK Biobank and 1000 Genomes Project.
Timing and spatial scale	These details are available from the official UK Biobank and 1000 Genomes Project websites.
Data exclusions	Low coverage samples were excluded.
Reproducibility	N/A
Randomization	UK Biobank target samples are allocated by their self-reported ethnic groups. 1000 Genome Project samples are allocated by their populations and continents.
Blinding	Blinding not possible.

Did the study involve field work? ☐ Yes ☒ No

Reporting for specific materials, systems and methods

We require information from authors about some types of materials, experimental systems and methods used in many studies. Here, indicate whether each material, system or method listed is relevant to your study. If you are not sure if a list item applies to your research, read the appropriate section before selecting a response.

Materials & experimental systems

n/a	Involved in the study
☒	☐ Antibodies
☒	☐ Eukaryotic cell lines
☒	☐ Palaeontology and archaeology
☒	☐ Animals and other organisms
☒	☐ Clinical data
☒	☐ Dual use research of concern
☒	☐ Plants

Methods

n/a	Involved in the study
☒	☐ ChIP-seq
☒	☐ Flow cytometry
☒	☐ MRI-based neuroimaging

Plants

Seed stocks	N/A
Novel plant genotypes	N/A
Authentication	N/A
